# Visual Binocular Disorders and Their Relationship with Baropodometric Parameters: A Cross-Association Study

**DOI:** 10.1155/2020/6834591

**Published:** 2020-08-04

**Authors:** María Carmen Sánchez-González, Estanislao Gutiérrez-Sánchez, Pinero-Pinto Elena, Carmen Ruiz-Molinero, Verónica Pérez-Cabezas, José-Jesús Jiménez-Rejano, Manuel Rebollo-Salas

**Affiliations:** ^1^Department of Physics of Condensed Matter, Optics Area, University of Seville, Reina Mercedes S/N, Seville, Spain; ^2^Department of Surgery, Ophthalmology Area, University of Seville, Doctor Fedriani S/N, 41009 Seville, Spain; ^3^Physiotherapy Department, Faculty of Nursing, Physiotherapy and Podiatry, University of Seville, C/Avicena S/N, 41009 Seville, Spain; ^4^Department of Nursing and Physiotherapy, Faculty of Nursing and Physiotherapy, University of Cadiz, C/Ana de Viya, 52, 11009 Cadiz, Spain

## Abstract

The aim of this study was to establish a relationship between nonstrabismic binocular dysfunction and baropodometric parameters. A total of 106 participants underwent binocular vision assessment by evaluating horizontal heterophoria, horizontal and vertical fusional vergence ranges, and vergence facility. Posturography was measured using the FreeMED baropodometric platform. Among the variables that the software calculates are foot surface, foot load, and foot pressure. Our results showed that in the participants with positive fusional vergence (PFV) (near) blur and recovery values outside the norm, there are statistically significant differences between the total foot area (*p* < 0.05), forefoot area (*p* < 0.05), forefoot load (*p* < 0.05), and rearfoot load (*p* < 0.05), in all of the cases of left foot vs. right foot. In the group of subjects who did not meet Sheard's criterion (distance), that is, those with unstable binocular vision, there was a statistically significant difference (*p* < 0.01) between maximum left and right foot pressure. In conclusion, our results establish a relationship between nonstrabismic binocular dysfunctions and some baropodometric parameters.

## 1. Introduction

In humans, posture can be defined as the body's position when the subject stands without moving with the feet parallel and without external forces other than gravity that influence his/her body [1]. Maintaining posture, balance, and head and eye movements result from afferent cervical information from the vestibular, visual, and proprioceptive systems reaching different parts of the central nervous system (CNS) [2, 3]. The vestibular system provides the brain information on the position and orientation of the head, and the somatosensory system through the mechanoreceptors reports on the position and orientation of the body. Vision influences head and body positions [4]. Subjects with altered binocular vision reported various symptoms [5] and changes in neck posture. This is due to the adaptation of the head to maintain binocularity and optimize visual acuity, which can cause musculoskeletal problems [6]. These three systems work together to control posture [7]. The visual system is responsible for most sensory perception, and many of our movements are controlled by our eyes [8].

Many investigations have established a relationship between vision and posture. Others affirmed the influence exerted by binocular vision on postural stability, and convergence seems to significantly reinforce postural control [9–13]. Nonstrabismic binocular dysfunctions differ, and their classification is important for the most appropriate treatment.

Wick [14] described a classification system for nonstrabismic binocular disorders based on the distance phoria (tonic vergence) and AC/A ratio (change in convergence caused by a certain change in accommodation). In this system, the possible diagnoses are divided into three main categories of binocular vision problems based on the AC/A ratio. Low AC/A ratio anomalies refer to convergence insufficiency (CI) and divergence insufficiency (DI); normal AC/A ratio is basic exophoria, basic esophoria, and fusional vergence dysfunction (FVD); and high AC/A ratio disorders include convergence excess (CE) and divergence excess (DE) [15].

Bucci et al. [9] reported that a group of children suffering from vertigo had a poor vergence range that caused changes in posture. Other studies have shown how vergence eye movements influence postural stability, claiming that it improves when the eyes converge to focus on a nearby object and worsens when the eyes look far away [10, 11]. Kapoula et al. [12] affirmed that changes in vergences can cause vertigo, imbalance, and postural instability. They studied a group of subjects with bilateral idiopathic loss of vestibular function and found that convergence was affected in all of them. However, these patients' posture improved when they were focused on a nearby point. Zhang et al. [13] described a group of children who presented reduced ranges of horizontal vergence in both directions, convergence and divergence, and twisted their heads in an attempt to improve their binocular vision.

Other studies report significant improvements in postural control after strabismus surgery [1, 8, 16]. They suggest that these were not due to the restoration of binocular vision, but rather the realignment of the visual axes that facilitates the visual perception and proprioception of the extraocular muscles [17, 18], improving the postural stability.

In the consulted bibliography, we found studies that established relationships between strabismus, binocular dysfunctions, and posture. However, none identified the type of binocular changes. The most widely used classification system in optometry includes several possible diagnoses: convergence insufficiency (CI), divergence insufficiency (DI), convergence excess (CE), divergence excess (DE), fusional vergence dysfunction (FVD), basic exophoria, basic esophoria, and vertical dysfunction [19].

In our study, we thoroughly evaluated the subjects' binocular vision by determining the horizontal heterophoria values and range of horizontal vergences in both directions using base-in (BI) and base-out (BO) vergence facility testing (VF) [20]. Our objective was to identify nonstrabismic binocular dysfunction that may be present in the study population and its possible relationship with baropodometric parameters.

## 2. Materials and Methods

### 2.1. Design

An analytical, observational, cross-sectional, prospective, cross-association study was conducted at the faculty of pharmacy at the optics and optometry facilities at the University of Seville.

### 2.2. Ethics

This study followed the tenets of the Declaration of Helsinki. Informed consent was obtained from the subjects after explaining the nature and possible consequences of the study. The Institutional Review Board of the University Hospital Virgen Macarena of the University of Seville approved the research.

### 2.3. Subjects

The sample was composed of 106 subjects (58 women and 48 men). The participants' mean age was 38 ± 14 (18-62) years, and the sample's mean body mass index was 25.91 ± 4.79 (17.49-38.48). The selected population was comprised of students, professors, and administration and service staff from the University of Seville. The inclusion criteria included age between 18 and 70 years old. All of the subjects had at least 20/20 best-corrected visual acuity and the absence of ocular motility defects, strabismus, nystagmus, amblyopia, or any ocular or systemic disease that could affect the results. Subjects who had undergone some type of ocular surgery or had a history of head trauma, cervical fracture, or surgery in this area and those with intellectual disabilities or who suffered any type of degenerative disease or neurological disorder were excluded.

The sample size was determined using the G ∗ Power 3.1.9.4 program. The following data were considered: an alpha error of 0.05, a study power of 80%, two-tailed hypotheses, and an effect size for the relationship between the variables and baropodometric parameters *R*^2^ of 0.092 obtained from a previously published study [21]. We used these data to obtain a sample size of 80 subjects. A total of 106 subjects were included.

### 2.4. Measurements

#### 2.4.1. Optometry Assessment

The following binocular function variables were measured:

- Sheard's criterion: This indicates that the blur value in the opposite vergence must be greater than or equal to twice the heterophoria. If the Sheard criterion is not met, we can assume that the patient will present visual symptoms [22]
- Horizontal heterophoria (prism diopters, Δ): This represents the degree of misalignment of the visual axes. It was measured at a distance (6 m) and near (40 cm) with an occluder, prism bar, and accommodative target [23]
- Horizontal fusional vergences (prism diopters, Δ): The amplitude of both the positive (convergence) and negative fusional vergences (divergence) was measured using a phoropter's rotary prism (ESSILOR MPH100E S/N000104 phoropter). The patient had to indicate when they saw blurred text (blur point), double images (breakpoint), and the image again (recovery point) [24]
- Vertical fusional vergences (prism diopters, Δ): These were measured using the phoropter's rotary prisms (ESSILOR MPH100E S/N000104 phoropter). The patient had to indicate when they saw doubled text (breakpoint) and the image again (recovery point) [24]
- Vergence facility (VF) (cycles per minute (cpm)): This was quantified with a prismatic combination 3 Δ base-in (BI)/12 Δ base-out (BO) [25]

#### 2.4.2. Baropodometric Procedure

Posturography was measured using the FreeMED baropodometric platform (Sensor Medica, Guidonia Montecelio, Rome, Italy). The platform size was 74 × 64 cm, with an effective surface of 60 × 50 cm and a thickness of 8 mm. The platform included 24 K gold sensors that provided high repeatability and reliability of measurements [26–33].

The data were recorded using FreeStep software version 1.4.01, which includes the same baropodometric platform. The participants were instructed to place their bare feet on a platform for bipodal support and in a natural and relaxed way, with their feet in a “physiological” position taking a few steps in the same place, with their heels aligned and 5 cm between them. The subjects were instructed to remain completely still for 7 seconds until the end of the examination. During the test, the subjects remained in an orthostatic position with their arms along the body and stared at a fixed point marked on the wall 2 m away at the height of each individual's glabella. Three consecutive records were obtained to calculate the mean of each of the static baropodometric parameters. All of the patients were wearing their optical correction devices during the measurements.

**Figure 1:**
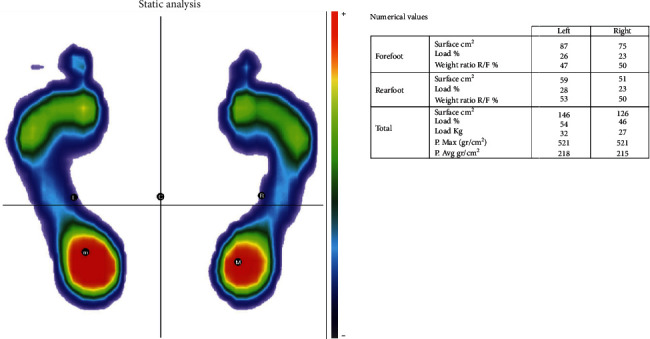
Static baropodometric parameters.

Computerized baropodometric analysis records plantar imprints and ground reaction forces during upright quiet standing [34]. The patient's foot pressure with associated numerical information was collected and displayed on the four quadrants of the foot (anterior, posterior, left, and right). This was achieved automatically using FreeStep software with the distal 60% of the foot length as the forefoot and the proximal 40% as the rearfoot. This enabled the determination of the percentage of weight supported by each foot and the symmetry ratio between them [35]. FreeStep calculated the following static baropodometric parameters (Figure 1):

1. Foot surface: This variable included the total surface and the surface of the forefoot and rearfoot for both feet expressed in square centimeters
2. Foot load: This variable included each foot's load and the load of the forefoot and rearfoot for both feet expressed as the percentage of weight supported by each foot and the symmetry ratio between them [35]
3. Foot pressure: This included the maximum and average pressure of both feet expressed in grams (g)/square centimeter

### 2.5. Data Analysis

The data were analyzed with SPSS 24 for Windows (SPSS Science, Chicago, IL, USA). The normality of our variables was verified with the Shapiro-Wilk test. A descriptive data analysis showed the number and proportion of each category in the qualitative variables and the mean and SD in the quantitative variables, the normally distributed variables, and the median and interquartile range (IQR: Q1-Q3) in the nonnormally distributed variables.

The relationship between the variables related to the subjects' binocular vision and those related to their posture was analyzed. The Pearson coefficient (*r*) value was determined, and simple and multiple (using the stepwise method) linear regression analyses were conducted showing the values of the coefficient of determination *R*^2^ and the nonstandardized coefficient *b*.

The variables' values related to posture were compared in the groups of subjects established according to the normative values of the variables that defined their binocular vision (inside and outside the norm) considered in isolation (intrasubject analysis). In these analyses, when the variables were adjusted to normal, we used Student's *t*-test for related samples, and for the variables that did not adjust to normal, the Wilcoxon signed-rank test was used. Then an intersubject analysis was conducted. The baropodometric parameter values of the differences between the left and right feet were compared in the subjects with the vergence function values within the norm vs. those that were outside the norm.

All of the statistical tests were conducted with a 95% confidence interval (CI) (*p* < 0.05).

## 3. Results

(Table 1 shows the variables' values that defined the participants' binocular vision and their classification as within or outside the normative values of these variables according to Scheiman and Wick's criteria [36]. (Table 2 shows the variables baropodometric. (Table 3 shows the correlations between variables and nonstandardized coefficients.

The intrasubject analysis demonstrated that in the participants outside the norm compared to those within the norm, in the variables that described their binocular function, there were greater differences in the distribution loads and pressures and on the surfaces of the left foot compared to the right and the forefoot compared to the rearfoot. The subjects outside the norm showed statistically significant differences or tendencies toward statistical significance for the following: (1) Sheard's criterion (distance) (*n* = 21) and maximum pressure on the left foot (672.36 ± 207.85 g/cm^2^) vs. the right foot (608.53 ± 178.74 g/cm^2^) with a *p* value < 0.01; (2) PFV (distance) blur (*n* = 23) and total left foot area (123.82 ± 34.93 cm^2^) vs. total right foot area (116.27 ± 35.24 cm^2^) with a *p* value < 0.05; (3) PFV (distance) blur (*n* = 23) and surface of the left forefoot (69.82 ± 20.85 cm^2^) vs. surface of the right forefoot (64.64 ± 22.60 cm^2^) with a *p* value < 0.05; (4) PFV (distance) blur (*n* = 23) and surface of the left rearfoot (54.09 ± 15.39 cm^2^) vs. surface of the right rearfoot (51.50 ± 14.17 cm^2^) with a *p* value = 0.05; (5) PFV (distance) recovery (*n* = 28) and surface of the left forefoot (74.15 ± 25.33 cm^2^) vs. surface of the right forefoot (70.59 ± 27.22 cm^2^) with a *p* value = 0.06; (6) PFV (distance) recovery (*n* = 23) and load of the left forefoot (52.00 ± 7.43%) vs. load of the right forefoot (50.22 ± 8.62%) with a *p* value < 0.05; (7) PFV (distance) recovery (*n* = 28) and load of the left rearfoot (48.00 ± 7.43%) vs. load of the right rearfoot (49.78 ± 8.62%) with a *p* value < 0.05. The subjects within the normative values did not present differences in these variables, with the exception of one variable, (8) lateral phoria (distance) (*n* = 90) and left rearfoot (58.37 ± 15.63 cm^2^) vs. right rearfoot (57.04 ± 16.34 cm^2^) with a *p* value = 0.060.

The subjects outside the norm presented statistically significant differences or with a tendency toward statistical significance for the following: (1) NFV(near) recovery (*n* = 27) and load of the left forefoot (52.48 ± 7.40%) vs. load of the right forefoot (50.96 ± 7.79%) with a *p* value = 0.063; (2) NFV (near) recovery (*n* = 27) and load of the left rearfoot (47.52 ± 7.40%) vs. load of the right rearfoot (49.04 ± 7.79%) with a *p* value = 0.06; (3) PFV (near) blur (*n* = 50) and left forefoot load (51.78 ± 6.63%) vs. left rearfoot load (48.22 ± 6.63%) with a *p* value = 0.063; (4) PFV (near) recovery (*n* = 15) and left rearfoot surface (55.27 ± 13.23 cm^2^) vs. right rearfoot surface (51.67 ± 16.27 cm^2^) with a *p* value = 0.066; (5) PFV (near) recovery (*n* = 15) and left forefoot load (54.60 ± 6.29%) vs. right forefoot load (57.53 ± 8.84%) with a *p* value = 0.057; (6) PFV (near) recovery (*n* = 15) and left rearfoot load (45.40 ± 6.29%) vs. right rearfoot load (42.47 ± 8.84%) with a *p* value = 0.057; (7) PFV (near) recovery (*n* = 15) and left forefoot load (54.60 ± 6.29%) vs. left rearfoot load (45.40 ± 6.29%) with a *p* value = 0.013; (8) PFV (near) recovery (*n* = 15) and right forefoot load (57.53 ± 8.84%) vs. right rearfoot load (42.47 ± 8.84%) with a *p* value = 0.005. The participants within the norm did not present differences in these variables, with the exception of (9) NFV (near) break (*n* = 60) and left rearfoot surface (56.85 ± 13.07 cm^2^) vs. right rearfoot surface (54.83 ± 14.61 cm^2^) with a *p* value = 0.016 and (10) NFV (near) recovery (*n* = 78) and left rearfoot surface (56.71 ± 15.08 cm^2^) vs. right rearfoot surface (55.13 ± 15.73 cm^2^) with a *p* value = 0.037.

Significant differences were found only between the subjects within and outside the norm in the absolute value of the difference between the left and right feet in the variables PFV near recovery and PFV distance recovery. In the first of these variables, the subjects within the norm had a lower value of the difference in the load between both forefeet (median = 0.0, Q1 = −3.0, and Q3 = 3.0) compared to the subjects outside the norm who had a higher imbalance in their left loads (median = −2.0, Q1 = −6.0, and Q3 = 0.5) with a *p* value = 0.04. This also occurred with the difference in the load between both setbacks, with the subjects outside the norm presenting greater differences (median = 2.0, Q1 = −5.0, and Q3 = 6.0) than those within the norm (median = 0.0, Q1 = −3.0, and Q3 = 3.0) with a *p* value = 0.04. In the second PFV distance recovery variables, there were greater differences in the forefoot load in the subjects outside the norm (median = 2.0, Q1 = −1.5, and Q3 = 4.5) than in those within the norm (median = −1.0, Q1 = −3.0, and Q3 = 2.0) with a *p* value = 0.03 and in the rearfoot load in the participants outside the norm (median = −2.0, Q1 = −4.5, and Q3 = 1.5) than in those who were inside the norm (median = 1.0, Q1 = −2.0, and Q3 = 3.0) with a *p* value = 0.03. In the differences in the loads within the same foot, in the variable PFV near recovery, in both the left and right foot, the subjects outside the norm had a greater load in the forefoot (forefoot-rearfoot left load difference mean = 9.2, SD = 12.58; forefoot-rearfoot right load difference mean = 15.1, SD = 17.7), while those within the norm showed a more balanced load distribution between the forefoot and rearfoot (forefoot-rearfoot left load difference mean = 0.3, SD = 13.8; forefoot-rearfoot right load difference mean = −1.5, SD = 15.6). These differences were statistically significant (forefoot-rearfoot left load difference *p* = 0.02; forefoot-rearfoot right load difference *p* = 0.002).

## 4. Discussion

This study fully evaluated the participants' binocular vision to determine the presence of nonstrabismic binocular dysfunctions and analyze whether there was a relationship between the visual system and posture through a correct and balanced footfall.

The results of this work are in line with previous studies demonstrating the relationship between the visual system and posture [1, 9–12, 37]. The study subjects with altered binocular vision demonstrated changes in their baropodometric parameters, which could cause postural alterations.

In the present study, binocular vision was assessed by evaluating Sheard's criterion, horizontal heterophoria, horizontal and vertical fusional vergence ranges, and vergence facility. These variables determined the relationship between the presence of nonstrabismic binocular dysfunctions with the baropodometric variables. By analyzing the relationships, considering the normative values of the variables that defined the subjects' binocular vision, in the group of subjects who did not meet Sheard's criterion (distance), that is, those with unstable binocular vision [22], there was a statistically significant difference (*p* < 0.01) between the maximum left and right foot pressure.

However, the subjects with PFV (near) blur and recovery values outside the norm, a situation that determines altered ranges of horizontal fusion amplitude associated with deviations [31, 32], there are statistically significant differences between the total foot area (*p* < 0.05), forefoot area (*p* < 0.05), forefoot load (*p* < 0.05), and rearfoot load (*p* < 0.05) in all of the cases of left foot vs. right foot. In the participants with NFV (near) recovery outside the norm, a sign that characterizes the presence of excess convergence [5], differences appeared between the forefoot load and left vs. right rearfoot load with a value (*p* = 0.063). We identified the subjects with PFV (near) blur and recovery values out of the norm, which determined altered horizontal fusion amplitude ranges and were associated with convergence insufficiency (CI) [38–40]. In these participants, differences appeared between the distribution of loads and the forefoot and rearfoot surface of the same opposite foot or feet. In no cases were the differences statistically significant. There was a statistical difference (p = 0.013) in only 15 subjects with altered PFV (near) recovery values between the left forefoot load and left rearfoot load [38–40]. In these participants, differences appeared between the distribution of loads and forefoot and rearfoot surface of the same opposite foot or feet. In no cases were the differences statistically significant.

Binocular vision uses both eyes that move simultaneously due to the extraocular muscles, which control convergence and divergence and are responsible for binocular vision [24, 41]. Binocular vision disorders can generate postural adaptations to maintain binocularity and achieve visual comfort. In a literature review, we found authors who affirmed the influence that binocular vision has on posture [16, 37, 42–46]. Prior research concluded that convergence seems to significantly enhance postural stability [10–12]. Maintenance of posture, balance, and head and eye movements result from afferent cervical information from the vestibular, visual, and proprioceptive systems that reaches different parts of the CNS [2, 3, 16]. The receptors involved in proprioception are mechanoreceptors located in the muscles, tendons, and joints. Mechanoreceptors are present in both the cervical region [47] and extraocular musculature (EOM) [17, 18].

Fox highlighted the role of proprioceptive signals from the extraocular muscles in postural stability [48]. In the dark, the body swaying with the eyes open is less stable than with the eyes closed due to the extraocular muscle tone. Legrand et al. [16] affirmed that after strabismus surgery, proprioception of the extraocular muscles improves, stabilizing the body. Three reflexes [49] that influence postural, head, and eye stability depend on cervical input: the cervical-colic reflex (CCR), cervical-ocular reflex (COR), and tonic neck reflex (TNR). These reflexes function together with others and are influenced by the vestibular and visual systems to coordinate the stability of the head, eyes, and posture.

Peterka [50] and Friedrich et al. [51] affirmed that when the visual or vestibular systems change, compensation mechanisms are produced in the other sensory systems. In this sense, the presence of binocular dysfunction could modify the tone of the extraocular muscles and proprioceptive information [52], altering the balance between the CCR, COR, and TNR reflexes, changing the stability and posture. Inadequate posture misaligns the human body. The feet are the foundations of the entire body, and changes in posture will cause incorrect and unbalanced footprints, since the body's weight will not be uniform or perfectly distributed via plantar support, altering the baropodometric parameters. In our study, the subjects had larger left lower limbs, possibly due to asymmetry that can be considered normal. It was previously demonstrated that measurable asymmetry is present even in healthy people with no known reasons for asymmetry [53–55]. A study by Overmoyer and Reiser [56] of healthy and active adults (20 subjects, 9 men and 11 women; mean ± SD: age = 21.9 ± 2.6 years, height = 171 ± 8.8 cm, and mass = 67.2 ± 1.9 kg) showed that there were asymmetries, not only in the length of the lower extremities but also in joint flexibility, bilateral asymmetries in flexibility, and bilateral asymmetries in the performance of balance tests and that were not related to injuries. Other factors that influence posture and determine possible asymmetry in the lower limbs are muscle strength and the range of motion of plantar flexion of the ankle, which also directly influence the ability to control static balance [57]. A combination of these factors could have influenced the results obtained in our sample in relation to the differences between the lower limbs.

### 4.1. Strengths, Limitations, and Perspective

This study's limitations include the large age range and the laterality of each subject. In future research, we will take into account laterality in relation to vision and limit the study by age to determine the possible effects of vision and the baropodometric variables in relation to age.

Given the relationship between vision and posture, future research should propose an intervention through a visual therapy program in subjects with nonstrabismic binocular dysfunctions because visual therapy has proven to be a useful treatment option in subjects with visual dysfunctions [58].

## 5. Conclusions

In conclusion, our results establish a relationship between nonstrabismic binocular dysfunctions and some baropodometric parameters. More research is needed to further identify this relationship. We also plan to include baropodometric variables of dynamics and stabilometry related to laterality.

### 5.1. Contribution to Health Care

Changes in the visual system and musculoskeletal disorders are important public health problems that affect considerable proportions of the general population, at work and in their daily and social lives. New technologies are available to assess visual and neck/shoulder musculature symptoms. This abnormal situation produces prolonged activation of the extrinsic and intrinsic muscles of the eye with distortion and imbalance in visual behavior resulting in nonstrabismic binocular dysfunctions [6]. This must be taken into account by primary health care professionals; visual dysfunctions may be the cause of musculoskeletal disorders. This study demonstrated the relationship between binocular vision dysfunction and changes in plantar support.

This study used a multidisciplinary approach that included several health professionals: ophthalmologists, optometrists, podiatrists, physiotherapists, and nurses. Patients with postural control problems should be evaluated visually and vice versa. This study demonstrated that patients cannot be observed from a single perspective. Therefore, a multidisciplinary approach is necessary for the care, treatment, and monitoring of pathologies associated with the optometric and baropodometric parameters.

## Figures and Tables

**Table 1:** Characteristics of the variables that defined binocular vision.

Distance				Near			
Variable		Mean ± SD	Inside the norm (%)	Variable		Mean ± SD	Inside the norm (%)
Sheard's criterion (*n* = 104)		—	83 (78.3)	Sheard's criterion (*n* = 104)		—	49 (46.2)
Lateral phoria, Δ (*n* = 106)		0.54 ± 2.25 X	91 (85.8)	Lateral phoria, Δ (*n* = 106)		−5.74 ± 6.92 X	56 (52.8)
NFV, Δ	Break (*n* = 105)	8.45 ± 2.27	88 (83.0)	NFV, Δ	Blur (*n* = 82)	10.98 ± 4.58	45 (42.5)
					Break (*n* = 106)	17.20 ± 5.16	60 (56.6)
	Recovery (*n* = 105)	4.48 ± 1.85	94 (88.7)		Recovery (*n* = 106)	11.53 ± 4.71	78 (73.6)
PFV, Δ	Blur (*n* = 73)	10.01 ± 4.56	50 (47.2)	PFV, Δ	Blur (*n* = 78)	10.86 ± 5.80	27 (25.5)
	Break (*n* = 101)	16.15 ± 6.84	70 (66.2)		Break (*n* = 103)	16.94 ± 7.41	41 (38.7)
	Recovery (*n* = 101)	8.06 ± 4.55	73 (68.9)		Recovery (*n* = 103)	9.38 ± 5.87	88 (83.0)
Vergence facility (cpm)		Not described		Vergence facility (cpm) (*n* = 85)		9.51 ± 4.67	16 (15.1)

SD: standard deviation; *Δ*: prism diopters; X: exophoria; NFV: negative fusional vergence; PFV: positive fusional vergence; cpm: cycles per minute.

**Table 2:** Characteristics of the baropodometric variables.

Variable		Left foot	Right foot	*p* value
		Mean ± SD	Mean ± SD	
Foot surface (cm^2^)	Total	131.43 ± 40.81	129.52 ± 41.98	0.16
	Forefoot	74.32 ± 25.93	73.31 ± 26.25	0.26
	Rearfoot	57.12 ± 15.77	56.10 ± 16.68	0.12
Foot load (%)	Total	52.87 ± 5.08	47.13 ± 5.08	<0.01
	Forefoot	50.95 ± 6.94	50.95 ± 8.42	0.50
	Rearfoot	49.05 ± 6.94	49.41 ± 8.42	0.50
Foot pressure (g/cm^2^)	Maximum	683.16 ± 201.94	624.71 ± 174.33	<0.01
	Medium	313.47 ± 91.61	284.47 ± 86.09	<0.01

SD: standard deviation.

**Table 3:** Correlations between variables and their significance and nonstandardized coefficients (regression models).

Variable		*r*	*p* value	*R* ^2^	Unstandardized coefficient *b*
NFV near blur	Left foot load (%)	0.241	0.029	0.058	0.271
	Right foot load (%)	-0.241	0.029	0.058	-0.271
PFV distance blur	Right surface (cm^2^)	0.249	0.033	0.062	-2.170
	Right forefoot surface (cm^2^)	-0.238	0.043	0.057	-1.334
	Right rearfoot surface (cm^2^)	-0.251	0.032	0.063	-0.845
	Left foot load (%)	0.239	0.042	0.057	0.260
	Right foot load (%)	-0.239	0.042	0.057	-0.260
	Maximum left foot pressure (g/cm^2^)	0.241	0.040	0.058	11.284
	Maximum right foot pressure (g/cm^2^)	0.253	0.031	0.064	9.720
PFV distance recovery	Right forefoot load (%)	-0.228	0.022	0.052	-0.422
	Right rearfoot load (%)	0.228	0.022	0.052	0.422
Vertical vergence distance break	Right forefoot load (%)	0.229	0.018	0.052	2.135
	Right rearfoot load (%)	-0.229	0.018	0.052	-2.135
Vertical vergence distance recovery with maximum left foot pressure (g/cm^2^)		-0.210	0.031	0.044	-53.518
Vertical vergence near recovery	Right foot surface (cm^2^)	0.203	0.037	0.041	9.864
	Right forefoot surface (cm^2^)	0.205	0.035	0.042	6.248
	Maximum left foot pressure (g/cm^2^)	-0.255	0.008	0.065	-59.573

NFV: negative fusional vergence; PFV: positive fusional vergence.

## Data Availability

The database used to support this study's findings is restricted by data protection laws to protect patient privacy. The data are available from M.C. Sánchez González (msanchez77@us.es) to those who meet the criteria for access to confidential data.
